# The Knowledge, Attitudes, and Practices of Parents of Children Admitted to the Paediatric Emergency Department with Fever

**DOI:** 10.3390/healthcare14050638

**Published:** 2026-03-03

**Authors:** Sema Bayraktar, Gülay Türk, Ahmet Butun, Zeynep Olgac Tay

**Affiliations:** 1 Faculty of Nursing, Istanbul University, 34116 Istanbul, Turkey; 2 Child Health and Diseases, Provincial Health Directorate, 34116 Istanbul, Turkey; 3 Department of Nursing, Faculty of Health Sciences, Mardin Artuklu University, 47000 Mardin, Turkey; 4 Institute of Graduate Education, Mardin Artuklu University, 47000 Mardin, Turkey

**Keywords:** parents, child, fever, fever management, knowledge, attitude, practice, emergency department

## Abstract

Introduction: Fever is one of the most common reasons for Paediatric Emergency Department (PED) visits, often driven by parental anxiety and misconceptions about fever management. This study aimed to evaluate the knowledge, attitudes, and practices of parents regarding childhood fever to identify gaps and guide targeted educational interventions. Understanding parental behaviors is crucial for improving care outcomes and reducing unnecessary PED utilization. Methods: This study is a descriptive cross-sectional study. The sample of this study consists of a total of 440 parents of children admitted to the Paediatric Emergency Department (PED) with complaints of fever. Convenience sampling was used to select the participants. Data were collected using a questionnaire covering sociodemographics, a form surveying the parents’ fever knowledge and attitude, and the validated parents’ fever management scale (Turkish version). The data were analyzed using the SPSS 22.0 statistical program. Results: Most parents (95.5%) reported prior experience with childhood fever, yet 54.1% lacked a regular physician. Common fever detection methods included tactile assessment (56.4%) and thermometers (27.3%). Parental concern arose at 39 °C (48.6%). Cold applications (41.6%) and antipyretics (21.1%) were frequent interventions. The mean PFMS-TR score was high (34.97 ± 4.27), indicating elevated caregiver burden. Scores varied significantly by the child’s age (higher for infants, *p* = 0.044) and maternal education (higher for educated mothers, *p* = 0.008). Satisfaction with healthcare staff correlated with higher scores (*p* = 0.024). Negative correlations emerged between parental age, number of children, and fever management scores (*p* < 0.05). Conclusions: Parents exhibited high interventionist behaviors and persistent knowledge gaps, underscoring the need for targeted education programs. Educational programs targeting fever management, tailored to parental demographics and misconceptions, are essential. Healthcare providers, particularly pediatric nurses, should prioritize clear communication and evidence-based guidance to empower parents and reduce unnecessary healthcare burdens. Future research should expand to diverse geographic and cultural settings to enhance generalizability.

## 1. Introduction

Fever is a common clinical manifestation that accounts for a substantial percentage of Paediatric Emergency Department (PED) visits, creating a critical need for understanding parental perceptions and behaviors [1,2,3]. Parents are concerned by fever and feel anxious if they perceive the fever to be high [4,5]. In addition, parents are afraid of fever as an indicator of serious illness [2]. Parents of children with fever commonly use PEDs to alleviate their fears and worries [2]. The existing literature showed that a majority of parents fear potential harm from fever, often attributing it to severe conditions or believing in the necessity of immediate medical intervention [1,3]. Understanding the knowledge, attitudes, and practices of parents is pivotal for improving care outcomes and reducing unnecessary PED visits.

Parents need to have adequate knowledge regarding causes, symptoms, measurement methods, fever-reducing techniques, and the use of antipyretic medications in order to manage fevers safely and effectively. The literature indicates that parents have inadequate knowledge regarding fever management and resort to inappropriate methods to reduce fevers. The literature reported that 65% of parents had insufficient knowledge about measuring and evaluating fevers, and 45% used antipyretic medications in incorrect dosages [6]. The lack of knowledge regarding fever management not only affects children’s health outcomes but also creates an extra burden for the healthcare system. In this context, evaluating parents’ knowledge, attitudes, and practices regarding fever management is of great importance for both the protection of children’s health and the effective use of healthcare services. Raising awareness among parents could contribute to the prevention of unnecessary visits to healthcare services, reducing the burden on the healthcare system and leading to the more efficient use of economic resources [7].

Evidence suggests that parents continue to exhibit persistent gaps in understanding key aspects of fever management, often exacerbated by misconceptions regarding its implications. This misjudgment can lead to overestimations of the severity of the child’s condition, contributing to nonurgent visits to the PED [2,8,9]. Furthermore, research has explored how parental beliefs, shaped by cultural and ethnic backgrounds, can influence their approach to fevers [10]. Determining whether parents have accurate information about fever management is important for identifying and correcting misconceptions and practices. Therefore, this study aims to assess the knowledge, attitudes, and practices of parents of children admitted to the PEDs with fever. This may contribute to the planning of educational programs for parents, helping them adopt more conscious and effective approaches to fever management. By identifying specific misconceptions and attitudes, more effective interventions could be developed, emphasizing the influence of knowledge and attitudes on the decision-making process.

## 2. Materials and Methods

### 2.1. Study Design

This study is a descriptive cross-sectional study.

### 2.2. Settings, Participants, and Sampling

The setting of the study was the PED at a tertiary hospital with a 700-bed capacity, located in the southeast of Turkey. Participants were parents of children who visited the PED and received the least urgent code during triage. The triage system used the endorsed three-level pediatric emergency triage system (Green–Yellow–Red). “Least urgent” corresponds to the “Green” category, defined as stable patients without life-threatening conditions, who are generally expected to be evaluated within 60–120 min. Children triaged as “Yellow” (semi-urgent) or “Red” (urgent) were not included. The questionnaires were self-administered on paper, and the researcher was available to provide clarification if needed. The sample of this study consists of a total of 440 parents of children admitted to the PED with complaints of fever. Convenience sampling was used to select the participants. Although a formal a priori sample size calculation was not performed due to the use of convenience sampling, this study included all eligible and consenting parents who presented to the PED during the data collection period. The final sample of 440 participants is adequate for detecting moderate effect sizes in group comparisons and correlation analyses at a 95% confidence level, providing sufficient statistical power for the planned analyses.

### 2.3. Data Collection

Data were collected between 26 February 2025 and 29 March 2025. The researcher (Z.O.T.) invited participants to the study, and the questionnaire was given to those who agreed to participate. The data were collected using the questionnaire technique, with a data collection form consisting of 3 parts. The time to fill out the questionnaire took about 15 min.

### 2.4. Data Collection Forms

#### 2.4.1. Sociodemographic Information and PED-Related Question Form

This form was developed by the researchers (A.B., S.B., and Z.O.T) based on the current literature and their previous experiences [1]. The form consists of 22 questions, including the age of the child and parents, the gender of the child, the level of education of the parents, the number of children in the family, where they live, when they visit the PED, how many times they have visited the PED in the last year, the health complaint of the child, their waiting time in the PED, the parents’ perception of the urgency of their child’s health condition, satisfaction with the PED healthcare staff, satisfaction with the communication skills of the PED healthcare staff, satisfaction with the information provided by the PED healthcare staff, satisfaction with the care received in the PED, satisfaction with the time allocated for their child, and their future perception regarding whether they would visit the PED in the future if they their child has the same health complaints. Satisfaction variables were assessed using three response options (Yes/No/Partly).

#### 2.4.2. Parents’ Fever Knowledge and Attitude Form

This form was developed by the researchers (A.B., S.B., and Z.O.T) based on the current literature [11,12,13,14]. This form consists of 29 questions regarding the parents’ fever knowledge and attitudes. The 29-item questionnaire was developed by the research team based on a review of the relevant literature. Content validity was established through expert evaluation by specialists in pediatric nursing and emergency care, and necessary revisions were made. A pilot test was conducted with 10 parents to assess clarity and comprehensibility, and minor modifications were implemented. The 29-item questionnaire was designed to describe parental knowledge and attitudes regarding childhood fever. Because the instrument does not generate a composite or total knowledge score and has not undergone full psychometric validation, results were interpreted descriptively. Therefore, conclusions regarding parental knowledge were made cautiously and framed in terms of response distributions rather than quantified knowledge levels.

#### 2.4.3. Parents’ Fever Management Scale—Turkish Version (PFMS-TR)

This scale aims to measure parents’ practices regarding childhood fever management. This scale was developed by Walsh et al. [15], and Altun et al. assessed the Turkish version’s validity and reliability [16]. The Turkish version of this scale was used in this study. The Turkish version of this scale consists of 8 items and is based on a 5-point Likert scale, ranging from 1 = never to 5 = always. The scale score ranges from 8 to 40, with a higher score indicating a greater caregiving burden on the parent in the presence of fever in the child [12]. In the original study of this scale, Cronbach’s alpha reliability coefficient was 0.80. In this study, the reliability of the fever management scale was found to be high, with a Cronbach’s alpha of 0.856.

### 2.5. Data Analysis

The data obtained in the study were analyzed using the SPSS 22.0 statistical program (IBM SPSS Statistics for Windows, Version 22.0, IBM Corp., Armonk, NY, USA). Frequency and percentage analyses were used to determine the descriptive characteristics of the participants, and mean and standard deviation statistics were used to examine the scale. Kurtosis and skewness values were analyzed to determine whether the research variables exhibited normal distributions. In the relevant literature, it is accepted that the results of the kurtosis and skewness values of the variables are between +1.5 and −1.5 [17] and +2.0 and −2.0 [18] for a normal distribution. It has been determined that the variables show a normal distribution. Parametric methods were used in the analysis of the data. To examine the differences in scale levels according to the descriptive characteristics of the participants, independent-groups *t*-tests and one-way analysis of variance (ANOVA) were used. When the overall ANOVA results were statistically significant, post hoc pairwise comparisons were conducted using Tukey’s Honestly Significant Difference (HSD) test to control for family-wise Type I errors.

### 2.6. Ethical Considerations

Ethical approval was obtained from the Mardin Artuklu University Non-Invasive Clinical Research Ethics Committee (Date: 18 February 2025; Reference number: 2025/2-2). Institutional permission was granted by the Provincial Directorate of Health (Date: 25 February 2025; Reference number: E-68051626-770-269432348). Written informed consent was obtained from all participants. This study was carried out in accordance with the principles of the Declaration of Helsinki.

## 3. Results

The descriptive characteristics of the participants are presented in Table 1. When examining the results, it is observed that the age distribution of the children was mostly in the range of 1–5 years (48.9%). It has been determined that the vast majority of participants were mothers (66.4%). While the highest rate of maternal education level was high school graduates (30.0%), the most common education level for fathers was undergraduate (38.2%). Fathers had a higher proportion of undergraduate education compared to mothers, while mothers were more frequently high school graduates. This distribution may reflect regional socio-cultural patterns in educational attainment. The mean age of mothers was 32.68 ± 6.23 (min = 19; max = 50), the mean age of fathers was 36.61 ± 7.15 (min = 21; max = 70), the mean number of children was 2.51 ± 1.32 (min = 1; max = 8), and the average waiting time in the PED was 42.70 ± 66.65 min (min = 1; max = 960). These results indicate that there is a wide diversity in the demographic characteristics and healthcare experiences of individuals caring for pediatric patients.

Some variables regarding the parents’ experiences with the PED are provided in Table 2. Out of the participants, 25% (n = 110) visited the PED 4–5 times in the last year. When looking at children’s health complaints for PED visits, the most common complaint was fever (68.6%), followed by upper respiratory tract infections (17.3%). Other complaints were less frequent and were grouped as gastrointestinal symptoms (5.2%), respiratory symptoms (3.4%), and other causes (5.5%). It has been determined that the participants assessed their children’s condition as urgent (44.5%) and semi-urgent (43.2%), while 12.3% perceived the condition as very urgent. Of the participants, 79.1% were satisfied with the healthcare staff. Similarly, 76.6% of parents expressed their satisfaction with the communication of healthcare staff. In addition, 71.4% of parents reported that they were adequately informed regarding their child’s condition. Out of the parents, 76.6% were satisfied with the care provided by healthcare staff, and 76.6% of parents indicated that they were satisfied with the time that was allocated to their children. Out of the parents, 89.3% (n = 393) reported that they could revisit the PED in the next year.

Table 3 presents the distribution of parental knowledge, attitudes, and practices regarding childhood fever. While most parents reported previous experience with fever and ownership of a thermometer, variability was observed in fever assessment methods and fever management practices. When the results were examined, the rate of parents who do not have a regular physician was 54.1%, constituting the majority. In total, 95.5% of the participants stated that their children had had a fever before, while 41.1% expressed that their child had a history of hospitalization. Among the methods parents use to understand that their children have a fever, the most common approach is “touching with hands” at 56.4%, followed by checking with a thermometer at 27.3% and attempting to understand by kissing the child’s forehead at 16.4%. The temperature of fevers that caused concern was mostly expressed as 39 °C (48.6%). It was found that 74.5% of the participants had a thermometer at home, with the most common measurement area being the forehead (60.2%), followed by the armpit (32.0%) and orally (4.5%).

It has been observed that parents mostly obtain information about fevers from healthcare staff (51.6%) and from family, friends, and neighbors (30.2%). The most commonly used method to reduce fevers is cold application (41.6%). In terms of the frequency of monitoring the fever, 44.8% reported that they measure it every 15–30 min. Among the reasons parents bring their children to the PED, “accompanying other complaints” (36.4%) was the most common reason, followed by “very high fever” (22.7%) and “prolonged fever” (15.2%). As a dosage determination method, the majority (80.2%) of participants rely on the physician’s recommendation. However, 6.6% make their decision based on medication information leaflets, and 7.7% based on the pharmacist’s recommendation. The percentage of those who report using antibiotics without a physician’s recommendation was 10.5%, while 77.3% use antibiotics based on a physician’s recommendation. While the proportion of those who perceived fever as a disease was 33.6%, 58.2% consider it a symptom of a disease. These results indicate that there were significant differences in the knowledge levels and practices of parents, and that evidence-based practices need to be supported. In particular, the guidance of healthcare staff and educational programs directed at the community are of great importance in this regard.

Fever management score averages are provided in Table 4. The average total score for fever management among participating parents was found to be 34.97 ± 4.27. The fact that the obtained mean value was very close to the upper limit indicates that the participants exhibit a high level of interventionist behaviors when their children have a fever.

Table 5 summarizes differences in fever management scores by sociodemographic characteristics and satisfaction variables. Significant differences were observed regarding the child’s age, the mother’s educational level, and satisfaction with healthcare staff, whereas most other sociodemographic variables were not significantly associated with fever management scores. According to the results, fever management scores show a significant difference in terms of the child’s age (*p* = 0.044). According to post hoc analysis results, the scores of parents with children under 1 year old were significantly higher than those of parents with children between 1–5 years old and those with children aged 11 and over. A statistically significant difference in the mother’s educational level has been found (*p* = 0.008). The scores of mothers with high school and bachelor’s degrees were significantly higher compared to those of mothers with a secondary school education. However, no significant differences were observed in variables such as the father’s educational level, parents’ employment status, and place of residence (*p* > 0.05).

When comparing groups based on satisfaction with healthcare staff, it was found that the fever management scores of parents who reported being satisfied with healthcare staff were significantly higher than those who were not satisfied (*p* = 0.024). Similarly, a significant difference was found in satisfaction with the communication of healthcare staff (*p* = 0.003); the scores of parents who reported being satisfied with communication were significantly higher than those who were not satisfied and those who were partially satisfied. No statistically significant difference was found in the scores based on satisfaction with the information provided by healthcare staff, as well as the adequacy of time allocated for care (*p* > 0.05). According to the correlation analysis, weak but significant negative relationships were found between the mother’s age (r = −0.161, *p* = 0.001), father’s age (r = −0.155, *p* = 0.001), and number of children in the family (r = −0.111, *p* = 0.020) and the fever management score.

## 4. Discussion

This study aimed to evaluate the knowledge, attitudes, and practices of parents regarding fever management in children. This study found that parents previously used the PED due to their children having a fever. The mean total score of parents for fever management was close to the upper limit of the scale, indicating that they have a high level of caregiving burden in situations where their children are febrile and exhibit anxiety-based behaviors. This interpretation aligns with the conceptual structure of the PFMS-TR. As described in the original development and validation studies of the scale, PFMS assesses parental anxiety, perceived burden, and intervention-oriented responses to childhood fever rather than objective knowledge alone. Therefore, higher scores reflect increased caregiver burden and interventionist tendencies, consistent with the instrument’s theoretical framework [15,16]. Similar studies have also found that parents become worried when their child has a fever [2,19,20,21]. This situation overlaps with what is often referred to in the literature as “fever phobia”, characterized by excessive protective and intervention behaviors, despite a significant awareness of fever among parents. Parents seek emergency care due to this state of anxiety [22]. In this study, as the educational level of the mother increased, the scale scores increased. This result shows that as the mother’s education level increases, parents approach their children’s febrile conditions in a more interventionist and cautious manner. However, no significant difference was observed in terms of variables such as the father’s education level, the working status of the mother and father, and the place of residence. Contrary to the results of this study, some studies found that parents’ knowledge and management of fever have been found to be inadequate, and this situation has been shown to be more related to parental characteristics (education level, socioeconomic status, etc.). The existing literature has noted that the educational level of parents is a determining factor in their knowledge of fever management, emphasizing that families with lower educational levels exhibit greater information deficiencies and anxiety [22,23,24]. In line with the existing literature [25], this study indicated that mothers are particularly stressed in situations concerning their children’s health. During hospital processes, the levels of anxiety experienced by mothers and fathers were found to vary despite their different educational levels. It was determined that 66.4% of the participants in this study were mothers. This result is consistent with the existing literature, which indicates that mothers feel a higher burden of care than fathers when it comes to hospital admissions related to their children [26].

The results of this study showed that parents’ scores regarding fever management in children vary significantly according to the age range of the child. A significant difference was found concerning the variable of the child’s age, with parents of children under 1 year old having higher fever management scores compared to other age groups. This situation indicates that more intense caregiving behaviors are exhibited towards younger children. A similar result was observed in the existing literature [27], where parents with younger children displayed more interventionist approaches towards fever and deviated from existing guidelines [27].

Although the parents were satisfied with the healthcare services and communication quality, it has been observed that the fever management scores were higher. [28]. The existing literature reported that parents of febrile children expressed significant concerns about complications such as brain damage and dehydration [29]. Research has indicated that when opportunities for interviews, videos, brochures, and clear information were provided, parental anxiety significantly decreases, and safe decision-making behaviors increase [30,31,32]. These results suggest that the high level of interventionism identified in the current study could be reduced through education programs targeted to parents.

In this study, as parental age and the number of children increased, the score for fever management decreased. The negative relationship found between the mother’s age, father’s age, and the number of children and the fever management score in the correlation analysis indicates that parents behave more cautiously as they gain experience. It can also be stated that, along with the average age of mothers being younger, they are more likely to access incorrect information online, which leads to more anxiety and, as a result, higher scale scores. It has also been shown that as age increases, this relationship does not always result in better management, and some misconceptions may be reinforced with age [33]. Therefore, accessing up-to-date and accurate information is as important as experience.

When the results of this study were evaluated as a whole, it was seen that some factors, such as the parents’ age, the child’s age, the mother’s education level, and healthcare service satisfaction, were effective in managing fever; however, many sociodemographic variables do not significantly affect this attitude. It is important that the education level and the quality of healthcare services positively influence parental behaviors; therefore, healthcare staff should play an active role in the information and communication processes aimed at parents.

### Strengths and Limitations

One of the strengths of our study was that it was conducted with a large sample of parents who have children with a fever. However, this study was conducted in a single tertiary hospital, so the data have limited the generalizability of the results. We recommend that future research should involve multiple hospitals across diverse geographic locations.

## 5. Conclusions

This study highlights the need to enhance parents’ understanding and attitudes toward fever management in children. Large-scale, sustainable educational initiatives to eliminate misconceptions about fever and to provide appropriate interventions for a febrile child could contribute to the management of fever in children. Pediatric nurses should be aware of misconceptions about fever and should organize educational programs aimed at educating parents, informing them about when to seek emergency care in cases of fever.

## Figures and Tables

**Table 1:** Descriptive characteristics of the participants.

Variables	n	%
**Age of the child**		
Younger than 1 year	25	5.7
1–5 years	215	48.9
6–10 years	132	30.0
11 years and older	68	15.5
**Gender of the child**		
Male	240	54.5
Female	200	45.5
**Relation to the child**		
Mother	292	66.4
Father	148	33.6
**Place of residence**		
City center	275	62.5
District	98	22.3
Village	67	15.2
**Parent’s Education Level**	**Mother n (%)**	**Father n (%)**
Primary school	100 (22.7)	53 (12.0)
Secondary school	95 (21.6)	74 (16.8)
High school	132 (30.0)	145 (33.0)
Bachelor’s degree and higher	113 (25.7)	168 (38.2)
**Parent’s Employment Status**	**Mother n (%)**	**Father n (%)**
Working	114 (25.9)	410 (93.2)
Not working	326 (74.1)	30 (6.8)
	**Mean**	**SD**
**Age of the mother**	32.680	6.230
**Age of the father**	36.610	7.147
**Number of children in the family**	2.510	1.316
**Waiting time in the PED (mins)**	42.700	66.652

**Table 2:** Parents’ experiences with the PED.

Variables	n	%
**Number of visits to the PED in the last 1 year**		
1	22	5.0
2–3	108	24.5
4–5	110	25.0
6–7	67	15.2
8–9	33	7.5
10 and more	100	22.7
**Health complaint of the child brought to the PED**		
Fever	302	68.6
Upper respiratory tract infection	76	17.3
Gastrointestinal symptoms (vomiting, stomachache)	23	5.2
Respiratory symptoms (cough, dyspnea, chest pain)	15	3.4
Other *	24	5.5
**Parent’s perception of urgency of the child’s condition**		
Non-urgent	190	43.2
Semi-urgent	196	44.5
Urgent	54	12.3
**Satisfaction with the healthcare staff**		
Yes	348	79.1
No	25	5.7
Partly	67	15.2
**Satisfaction with the communication of healthcare staff**		
Yes	337	76.6
No	28	6.4
Partly	75	17.0
**Satisfaction with information provided by healthcare staff**		
Yes	314	71.4
No	126	28.6
**Satisfaction with the care received by the child**		
Yes	337	76.6
No	36	8.2
Partly	67	15.2
**Adequacy of allocated time for care**		
Yes	337	76.6
No	41	9.3
Partly	62	14.1
**Thinking of revisiting the PED in the future for the same health problem**		
Yes	393	89.3
No	24	5.5
Partly	23	5.2

* Other: Allergy, headache, fatigue.

**Table 3:** Distribution of parents’ knowledge and attitude characteristics regarding fever.

Variables	N	%
**Regular doctor presence**		
Available	202	45.9
Not available	238	54.1
**Past history of fever**		
Yes	420	95.5
No	20	4.5
**Hospital admission**		
Yes	181	41.1
No	259	58.9
**Method of understanding fever**		
Touching with hand	248	56.4
Using a thermometer	120	27.3
Kissing child’s forehead	72	16.4
**Concerning fever range**		
37 °C	14	3.2
38 °C	128	29.1
39 °C	214	48.6
40 °C and higher	84	19.1
**Presence of a thermometer at home**		
Available	328	74.5
Not available	112	25.5
**Fever measurement area**		
Forehead	265	60.2
Armpit	141	32.0
Oral	20	4.5
Ear	10	2.3
Rectal	4	0.9
**Information source about fever**		
Healthcare staff	227	51.6
Family, friends, and neighbors	133	30.2
Television, radio, internet, and newspapers	80	18.2
**Fever reduction methods**		
Cold application	183	41.6
Showering with warm water	102	23.2
Giving an antipyretic	93	21.1
Showering with cold water	34	7.7
Wiping with vinegar, cologne, or alcohol	28	6.4
**Fever control frequency**		
0–15 min	92	20.9
15–30 min	197	44.8
30–45 min	105	23.9
45–60 min	46	10.5
**Reason for bringing the child to the PED**		
Due to the accompanying other complaints	160	36.4
The fever was very high	100	22.7
Prolonged fever	67	15.2
Could not alleviate the fever	61	13.9
Fear of feverish illness	52	11.8
**Dose decision methods**		
By physician’s recommendation	353	80.2
By nurse’s recommendation	4	0.9
Medication information leaflet	29	6.6
By pharmacist’s recommendation	34	7.7
With my own information	16	3.6
By the recommendation of someone I know	4	0.9
**Use of antibiotics without a physician’s recommendation**		
Yes	46	10.5
No	340	77.3
Sometimes	54	12.3
**Is fever a disease?**		
Yes, it is a disease	148	33.6
It is a symptom of a disease	256	58.2
No, it is not a disease	36	8.2

**Table 4:** Fever management score averages.

	n	Mean	SD	Min.	Max.	Kurtosis	Skewness	Scale Range
Fever management total	440	34.971	4.272	16.000	40.000	0.790	−0.893	8–40

**Table 5:** Differentiation of fever management scores according to descriptive characteristics.

Demographic Information	n	Fever Management Total
**Age of the child**		Mean ± SD
Younger than 1 year	25	36.76 ± 2.70
1–5 years	215	34.92 ± 4.25
6–10 years	132	35.20 ± 4.20
11 years and older	68	34.03 ± 4.77
F=		2.727
*p*=		**0.044**
PostHoc=		1 > 2, 1 > 4 (*p* < 0.05)
**Gender of the child**		Mean ± SD
Male	240	35.15 ± 4.31
Female	200	34.76 ± 4.22
t=		0.966
*p*=		0.335
**Relation to the child**		Mean ± SD
Mother	292	34.98 ± 4.16
Father	148	34.95 ± 4.49
t=		0.086
*p*=		0.932
**Mother’s education level**		Mean ± SD
Primary school	100	34.64 ± 4.91
Secondary school	95	33.86 ± 4.50
High school	132	35.69 ± 3.59
Bachelor’s degree and higher	113	35.35 ± 4.02
F=		3.955
*p*=		**0.008**
PostHoc=		3 > 2, 4 > 2 (*p* < 0.05)
**Father’s education level**		Mean ± SD
Primary school	53	34.83 ± 4.45
Secondary school	74	34.55 ± 4.64
High school	145	34.85 ± 4.15
Bachelor’s degree and higher	168	35.30 ± 4.16
F=		0.632
*p*=		0.595
**Employment status of mother**		Mean ± SD
Working	114	35.33 ± 4.30
Not working	326	34.84 ± 4.26
t=		1.054
*p*=		0.293
**Employment status of father**		Mean ± SD
Working	410	35.04 ± 4.18
Not working	30	34.07 ± 5.38
t=		1.201
*p*=		0.230
**Satisfaction with the healthcare staff**		Mean ± SD
Yes	348	35.19 ± 4.28
No	25	32.88 ± 4.57
Partly	67	34.60 ± 3.91
F=		3.766
*p*=		**0.024**
PostHoc=		1 > 2 (*p* < 0.05)
**Satisfaction with the communication of healthcare staff**		Mean ± SD
Yes	337	35.31 ± 4.17
No	28	32.75 ± 5.25
Partly	75	34.25 ± 4.04
F=		6.068
*p*=		**0.003**
PostHoc=		1 > 2, 1 > 3 (*p* < 0.05)
**Satisfaction with information provided by healthcare staff**		Mean ± SD
Yes	314	34.92 ± 4.38
No	126	35.10 ± 4.01
t=		−0.388
*p*=		0.687
**Adequacy of time allocated for care**		Mean ± SD
Yes	337	35.07 ± 4.24
No	41	34.39 ± 4.24
Partly	62	34.79 ± 4.49
F=		0.531
*p*=		0.588
		r/*p*
**Age of the mother**	r	−0.161 **
	*p*	0.001
**Age of the father**	r	−0.155 **
	*p*	0.001
**Number of children in the family**	r	−0.111 *
	*p*	0.020

F: ANOVA Test; t: Independent-groups *t*-Test; PostHoc: Tukey, HSD; * < 0.05; ** < 0.01; Pearson correlation analysis. Bold values indicate statistically significant results (*p* < 0.05).

## Data Availability

The datasets used and/or analyzed during the current study are available from the corresponding author upon reasonable request.

## References

[1] Butun A. (2024). The role of family health centres in preventing paediatric emergency department usage of parents of children with non-urgent conditions. BMC Primary Care.

[2] Butun A., Lynn F., McGaughey J., McLaughlin K., Linden M. (2022). Exploring attendance at emergency departments for children with non-urgent conditions in Turkey: A qualitative study of parents and healthcare staff perspectives. Emerg. Crit. Care Med..

[3] Hussain S.M., Al-Wutayd O., Aldosary A.H., Al-Nafeesah A., AlEed A., Alyahya M.S., Alfeneekh A.S., AlKadi S.A., Alghasham G.A., Aloyaidi G.A. (2020). Knowledge, Attitude, and Practice in Management of Childhood Fever Among Saudi Parents. Glob. Pediatr. Health.

[4] Kua P.H., Wu L., Ong E.L., Lim Z.Y., Yiew J.L., Thia X.H., Sung S.C. (2016). Understanding decisions leading to nonurgent visits to the paediatric emergency department: Caregivers’ perspectives. Singap. Med. J..

[5] Kelly M., Sahm L.J., Shiely F., O’Sullivan R., McGillicuddy A., McCarthy S. (2016). Parental knowledge, attitudes and beliefs regarding fever in children: An interview study. BMC Public Health.

[6] Walsh A.M., Edwards H.E., Courtney M.D., Wilson J.E., Monaghan S.J. (2005). Fever management: Paediatric nurses’ knowledge, attitudes and influencing factors. J. Adv. Nurs..

[7] Dinç S., Gözen D. (2023). Ailelerin Ateşe Yönelik Oral İlaç Uygulamalarının Geliştirilmesinde Hemşirenin Rolü. Sağlık Bilim. Üniversitesi Hemşirelik Derg..

[8] Kızılkan M.P., Özsezen B., Batu E.D. (2022). Factors Affecting Nonurgent Pediatric Emergency Department Visits and Parental Emergency Overestimation. Pediatr. Emerg. Care.

[9] Butun A., Linden M., Lynn F., McGaughey J. (2019). Exploring parents’ reasons for attending the emergency department for children with minor illnesses: A mixed methods systematic review. Emerg. Med. J..

[10] Shapiro D.J., Fine A.M. (2019). Patient Ethnicity and Pediatric Visits to the Emergency Department for Fever. Pediatr. Emerg. Care.

[11] Cinar N.D., Altun I., Altınkaynak S., Walsh A. (2014). Turkish parents’ management of childhood fever: A cross-sectional survey using the PFMS-TR. Australas. Emerg. Nurs. J..

[12] Akbayram H.T., Coskun E. (2020). Paediatric emergency department visits for non-urgent conditions: Can family medicine prevent this?. Eur. J. Gen. Pract..

[13] Ekim A., Üstünel T.B. (2024). Çocuklarda Ateş Yönetimine İlişkin Ebeveynlerin Bilgi ve Tutumlarının Değerlendirilmesi. Arel Üniversitesi Sağlık Bilim. Derg..

[14] Kılıç R., Kendir Ö.T., Gökay S.S., Çelik T., Özkaya A.K., Yılmaz H.L. (2016). Çocuklarda ateş ile ilgili ebeveynlerin tutum ve davranışları. J. Pediatr. Emerg. Intensive Care Med..

[15] Walsh A., Edwards H., Fraser J. (2008). Parents’ childhood fever management: Community survey and instrument development. J. Adv. Nurs..

[16] Altun İ., Cinar N.D., Walsh A. (2011). Psychometric properties of the parents’ fever management scale in a Turkish population. HealthMED.

[17] Tabachnick B.G., Fidell L.S. (2022). Using Multivariate Statistics.

[18] George D., Mallery P. (2019). IBM SPSS Statistics 25 Step by Step: A Simple Guide and Reference.

[19] Alsofyani B.A., Hassanien N.S. (2022). Factors affecting parent’s practice regarding the management of children’s fever. Cureus.

[20] Yiğit P., Sarıalioğlu A. (2022). Ateşli çocukların ebeveynlerinin kaygı düzeyleri ile ateş yönetimlerinin incelenmesi. J. Curr. Pediatr..

[21] Nijman R.G., Oostenbrink R., Dons E.M., Bouwhuis C.B., Moll H.A. (2010). Parental fever attitude and management: Influence of parental ethnicity and child’s age. Pediatr. Emerg. Care.

[22] Gulcan M.K., Sahiner N.C. (2023). Determining the fever-related knowledge and practices of mothers with children aged 1–5 years presenting to a child emergency service with fever complaints in Turkiye. J. Pediatr. Nurs..

[23] Barutcu A., Barutcu S. (2020). Evaluation of knowledge, attitudes and practices of parents presenting to a hospital emergency department with a complaint of fever in a child. Signa Vitae.

[24] Peetoom K.K., Ploum L.J., Smits J.J., Halbach N.S., Dinant G.-J., Cals J.W. (2016). Childhood fever in well-child clinics: A focus group study among doctors and nurses. BMC Health Serv. Res..

[25] Halldorsson B., Draisey J., Cooper P., Creswell C. (2018). Symptoms of social anxiety, depression, and stress in parents of children with social anxiety disorder. Br. J. Clin. Psychol..

[26] Missler M., Beijers R., Denissen J., Van Straten A. (2018). Effectiveness of a psycho-educational intervention to prevent postpartum parental distress and enhance infant well-being: Study protocol of a randomized controlled trial. Trials.

[27] Raffaeli G., Orenti A., Gambino M., Peves Rios W., Bosis S., Bianchini S., Tagliabue C., Esposito S. (2016). Fever and pain management in childhood: Healthcare providers’ and parents’ adherence to current recommendations. Int. J. Environ. Res. Public Health.

[28] Poirier M.P., Davis P.H., Gonzalez-Del Rey J.A., Monroe K.W. (2000). Pediatric emergency department nurses’ perspectives on fever in children. Pediatr. Emerg. Care.

[29] Karwowska A., Nijssen-Jordan C., Johnson D., Davies H.D. (2002). Parental and health care provider understanding of childhood fever: A Canadian perspective. Can. J. Emerg. Med..

[30] Chapron A., Brochard M., Rousseau C., Rousseau A.-C., Brujean M., Fiquet L., Gandemer V. (2018). Parental reassurance concerning a feverish child: Determinant factors in rural general practice. BMC Fam. Pract..

[31] Huang M.C., Shih C.J. (2004). Effects of fever education on changing parental concepts and management of fever in childhood. Hu Li Za Zhi J. Nurs..

[32] Broome M.E., Dokken D.L., Broome C.D., Woodring B., Stegelman M.F. (2003). A study of parent/grandparent education for managing a febrile illness using the CALM approach. J. Pediatr. Health Care.

[33] Purssell E. (2009). Parental fever phobia and its evolutionary correlates. J. Clin. Nurs..

